# Professional identity and mental health of rural-oriented tuition-waived medical students in Anhui Province, China

**DOI:** 10.1186/s12909-019-1603-1

**Published:** 2019-06-13

**Authors:** Ling Wang, Yuwei Yang, Jimin Zhu, Hong Xie, Chunxiao Jiang, Chi Zhang, Jie Li, Fen Huang

**Affiliations:** 1 Department of Epidemiology and Biostatistics, School of Public Health, Anhui Medical University, No. 81 Meishan Road, Shushan Districts, Hefei, 230032 Anhui China; 2 Department of Public Health and General Medicine, Anhui University of Chinese Medicine, 103 Meishan Road, Shushan District, Hefei, 230038 Anhui China; 3 School of Nursing, BengBu Medical College, 2600 East Sea Avenue, Longzi Lake District, Bengbu, 233030 Anhui China; 4 Department of Teaching Center for Preventive Medicine, School of Public Health, Anhui Medical University, 81 Meishan Road, Shushan District, Hefei, 230032 Anhui China; 5 Central Laboratory of Preventive Medicine, School of Public Health, Anhui Medical University, 81 Meishan Road, Shushan District, Hefei, 230032 Anhui China

**Keywords:** RTME, Medical students, Professional identity, Mental health

## Abstract

**Background:**

The shortage of primary care physicians in rural China is an enduring problem with serious implications for access to care. In response to the shortage in health workforce in rural areas, China government has launched the rural-oriented tuition-waived medical education (RTME) programme since 2010, aiming to train more general practitioners to address the rural health workforce requirements in middle and west areas. This study aims to investigate the prevalence of mental illness and the level of professional identity in the rural-oriented tuition-waived medical students (RTMSs), and to explore the impact of the RTMSs’ professional identity and related cognition and satisfaction with the RTME programme on mental health.

**Methods:**

We conducted a descriptive, cross-sectional study. A total of 1103 RTMSs and 1095 non-oriented medical students from seven medical universities (colleges) in Anhui province completed a demographic questionnaire, the Depression, Anxiety, Stress Scales and the Professional Identity Questionnaire for Undergraduate Students. Cognition and satisfaction with the RTME programme of the RTMSs were collected. Multiple linear regression analysis was used to analyze the data.

**Results:**

The prevalence of depression, anxiety, and stress in RTMSs were 11.8, 22.9 and 3.4%, respectively. The mean total scores of the Professional Identity Questionnaire for Undergraduate Students were 3.58 (SD = 0.61). Results of multiple linear regression model indicated that students who are dissatisfied with targeted primary healthcare institution are likely to suffer from depression and anxiety; moreover, students who enrolled in the rural-oriented tuition-waived medical education programme due to economic reason are more likely to suffer from anxiety. Furthermore, a significant positive correlation was found between professional identity and mental health.

**Conclusions:**

Providing better information about the RTME programme prior to enrollment and improving the students’ cognition of the policy’s effectiveness and the social value of rural healthcare work may contribute to improving the professional identity of the RTMSs. Meanwhile, a significant positive association was found between professional identity and mental health. This is a new perspective that shows that developing and improving professional identity may help medical students reduce the risk of psychological illness.

## Background

Despite decades of strategies designed to reduce the disparity in the supply of physicians between rural and urban areas, geographic maldistribution has remained a persistent feature of the physician workforce in China [1, 2]. Nearly three times as many healthcare professionals per 100,000 people practice in urban areas as in small rural areas. By contrast, approximately 43% of the population lives in rural China, and only approximately 12% of the physicians practice in rural locations [3]. The shortage of rural healthcare professionals has become a bottleneck that seriously restricts the further improvement of service and level of primary medical institutions.

In response to the shortage in health workforce in rural areas, China has proposed numerous health workforce development plans or policies, including the rural-oriented tuition-waived medical education (RTME) programme initiated and promoted since 2010. This programme aims to train additional general practitioners to address the rural health workforce requirements in the middle and west areas. In China, the three-tier health service delivery system consists of county hospitals, township health centers, and village clinics in rural areas, providing basic health care to the rural population. Township hospitals, as the main provider of rural primary healthcare services, play a critical role in serving the majority of approximately 590 million people in the rural areas of China [4–6]. However, the township hospitals are severely understaffed [7]. These hospitals have difficulty recruiting sufficient healthcare professionals or can employ only healthcare professionals with limited education. Therefore, the RTME programme was implemented. This programme aimed to enroll medical students mainly from rural areas to work in township hospitals for 6 years after they graduation. In this programme, the medical students would sign a contract with the medical training school and local health administrative department. The government would waive the related tuition fee for the students and provide them with a certain amount of living allowance during their studies in designated medical universities. Although the RTME programme has contributed considerably to the rural workforce in the past few years, a recent survey showed that only approximately 1.6% of graduates are willing to return to rural communities [8]. Obstacles that discourage them from practicing as a rural general practitioner include limited professional development prospect, unsound medical equipment and facilities, and inadequate financial remuneration, thereby simultaneously influencing the professional identity of RTMSs.

Physicians’ professional identity is usually defined as ‘the integration of the knowledge, skills, values, and behaviors of a doctor with one’s own unique identity and core values’ [9, 10].. Professional identity, or how a doctor thinks of himself or herself as a doctor, is considered to be a key factor in the ability of health professionals to provide high-quality care to improve patient outcomes [11, 12] and is believed to mediate the negative effects of a high-stress workplace [13] and improve clinical performance [14, 15] and job retention [16, 17]. A longitudinal cohort study conducted on the junior doctors, which is a critical period of transition from medical students to professional doctors, suggested that professional identity was significantly negatively related to burnout, including personal and work-related burnout. [18]. Thus, improved understanding of the professional identity of medical students provides an important contribution to improving mental health and career satisfaction in the professional practice.

Concerns surrounding mental health in the medical profession have been extensively discussed in recent years. The demands and pressure of medical training have been documented well to pose a huge challenge to the personal well-being of physicians [19]. These demands and pressures frequently lead to many negative consequences, such as poor academic performance [20], increased rates of substance use [21], and even suicide [22]. Thus, the psychological well-being of medical students is crucial not only for the medical students themselves, but also for the quality of health care they will provide and the doctor-patient relationship in the future.

For the present study, we aim to investigate all the RTMSs in Anhui Province to evaluate professional identity and mental health and identify potential influencing factors.

## Methods

### Study design and participants

This cross-sectional study was conducted among all RTMSs from seven medical universities (colleges) in Anhui Province using an anonymous self-administered questionnaire. For comparison, 1103 RTMSs and 1095 non-oriented medical students from the same universities (colleges) were invited to participate in this study. These universities (colleges) offer two education formats, namely, 3-year junior college study programme and 5-year Bachelor of Medicine degree programme. A bachelor’s degree study programme, which is divided into three-preclinical years followed by two clinical years. Similarly, the junior college study programme comprises of two pre-clinical years followed by one clinical year. This study has been reviewed and approved by the Ethics Committee of Anhui Medical University.

### Measure

#### Professional identity

The Professional Identity Questionnaire for Undergraduate Students (PIQUS) was compiled in a Chinese language by Qin Panbo to measure professional identity among the undergraduate students [23]. This instrument contains 23 items and yields scores on the following four dimensions: cognition (five items), emotionality (eight items), behavior (six items) and fitness (four items). Items are accessed on a 5-point Likert scale (“complete conformity” = 5, “conformity” = 4, “neutral” = 3, “inconformity” = 2, and “complete inconformity” = 1). The total scores are calculated by the sum of scores of each question divided by the total number of questions; high scores indicate high level of professional identity.

### Mental health

Mental health problems were measured using a validated Chinese version of the Depression Anxiety Stress Scale-21 (DASS-21). DASS-21 is a set of three self-administered subscales (seven questions each) designed to measure the negative emotional states of depression, anxiety, and stress. The 21-item instrument asked respondents to rate the presence of these items of a symptom over the past week on a 4-point Likert scale with scores from 0 to 3. The scores of each subscale is equal to the sum of seven corresponding items and ranges from 0 to 21. A high subscale score indicates an unfavorable psychological status. Previous studies have demonstrated that the Chinese version of DASS-21 has excellent psychometric properties, with an overall Cronbach’s alpha coefficient of 0.89 [24].

### Demographic characteristics, cognition and satisfaction with the RTME programme

Demographic factors include age, gender, education level, location of hometown, family monthly income, father’s occupation and education, and mother’s occupation and education. Cognition of the RTME programme consisted of the following questions: (1) why did you decide to enroll in the programme? (2) Do you understand the conditions of the policy before enrolling? (3) Do you understand the conditions of targeted primary health organization? (4) Do you understand the Healthy China 2030 Plan Outline? (5) Do you understand the equitable access to basic public health services? (6) What was your cognition of the RTME programme? Satisfaction with the RTME programme included four items as following: satisfaction with targeted primary health organization, six years’ service period, living allowance and standardized training.

### Data analysis

The statistical analysis was performed using SPSS for Windows (version 23.0; SPSS Inc., Chicago, IL, USA). All study variables were carried out with a descriptive analysis. Quantitative variables were described as mean ± standard error, whereas categorical variables were expressed as frequencies. In this study, the independent variables included demographic characteristics, cognition and satisfaction with the RTME programme; the dependent variables were professional identity and mental health. Multiple linear regression analysis was conducted to explore the factors affecting the professional identity. In Model 1, the impacts of the demographic characteristics were assessed. The factors of cognition of the RTME programme were added in Model 2, and the satisfaction with the RTME programme were further added in Model 3. Multivariate stepwise linear regression analysis was applied to access the factors associated with mental health. A *P*-value less than 0.05 was regarded as statistically significant.

## Results

### Demographic characteristics

The mean age of the RTMSs was 20.5 years old. The profile of the sample studied was predominantly female (56.5%) and family monthly income of more than 5000 yuan (19.1%). 84.7% of the RTMSs were from rural areas.. Within the non-oriented medical student group, fewer than half of the respondents were male (43.7%), and 31.2% of population had a family income of more than 5000 yuan per month. Furthermore, 67.4% of the non-oriented medical students were from rural areas. The other personal characteristics were listed in Table 1.

**Table 1:** Demographic characteristics of the participants

Demographic characteristic	No. (%) of Participants	
	RTMSs (*n* = 1103)	Non-oriented medical students (*n* = 1095)
Gender		
Male	478 (43.5)	479 (43.7)
Female	621 (56.5)	616 (56.3)
Educational level		
Undergraduate	724 (65.6)	720 (65.8)
Junior college	379 (34.4)	375 (34.2)
Academic years		
Pre-clinical	707 (64.1)	699 (63.8)
Clinical	396 (35.9)	396 (36.2)
Location of hometown		
Rural	934 (84.7)	738 (67.4)
Urban	162 (14.7)	357 (32.6)
Family monthly income (RMB)		
≤ 5000	887 (80.9)	751 (68.8)
> 5000	210 (19.1)	341 (31.2)
Father’s occupation		
Worker	313 (28.5)	305 (27.9)
Farmer	433 (39.4)	326 (29.9)
Businessman	177 (16.1)	220 (20.1)
Professionals	116 (10.5)	158 (14.5)
Others	61 (5.5)	83 (7.6)
Father’s education		
Primary school or below	251 (22.8)	180 (16.5)
Junior high school	534 (48.5)	531 (48.5)
Senior high school	224 (20.3)	229 (20.9)
Postsecondary or above	92 (8.4)	154 (14.1)
Mother’s occupation		
Worker	208 (19.0)	201 (18.4)
Farmer	535 (48.8)	404 (37.0)
Businessman	188 (17.1)	254 (23.2)
Professionals	54 (4.9)	96 (8.8)
Others	112 (10.2)	138 (12.6)
Mother’s education		
Primary school or below	523 (47.6)	421 (38.5)
Junior high school	413 (37.6)	411 (37.6)
Senior high school	126 (11.5)	175 (16.0)
Postsecondary or above	37 (3.4)	86 (7.9)

#### Cognition and satisfaction with the RTME programme

Table 2 summarized the cognition and satisfaction with the RTME programme of the RTMSs. Prior to enrolment, 13.2% of the RTMSs did not understand the policy conditions, whereas 36.3% of the RTMSs understood the policy very well or relatively well. The majority (44.4%) of the RTMSs selected the programme due to the recommendation of family members or teachers, whereas 17.6% selected the programme because of personal interests. In terms of the view on the RTME programme, 45.4% believed that this programme limits personal career development, and only 8.4% believed this programme will help achieve personal value. In terms of satisfaction with the RTME programme, 47.6% of the RTMSs were satisfied with targeted primary health organization, whereas 50.9% were satisfied with the living allowance; however, the percentage of satisfaction with 6 years’ service period was the lowest at only 34.0%.

**Table 2:** Cognition and satisfaction with the RTME programme of the RTMSs

Variable	No.(%) of the Participants
Reasons for enrolment in the RTME programme	
Recommendations of family members or teachers	489 (44.4)
Economic reasons	97 (8.8)
Serve the local people	102 (9.3)
Guaranteed employment	82 (7.4)
Personal interests	194 (17.6)
By mistake	137 (12.4)
Understanding the conditions of policy before enrolling	
Don’t understand	146 (13.2)
Generally understand	556 (50.5)
Very or relatively well	400 (36.3)
Understanding the conditions of targeted primary health organization	
Don’t understand	334 (30.3)
Generally understand	561 (50.9)
Very or relatively well	207 (18.8)
Understand the Healthy China 2030 Plan Outline	
Yes	91 (8.3)
No	1011 (91.7)
Understand the equitable access to basic public health services	
Yes	245 (22.2)
No	858 (77.8)
View on the RTME programme	
Limiting personal career development	501 (45.4)
Have a stable work	436 (39.5)
Achieve personal value	93 (8.4)
Other	73 (6.6)
Satisfaction with targeted primary health organization	
Yes	525 (47.6)
No	578 (52.4)
Satisfaction with six years’ service period	
Yes	375 (34.0)
No	728 (66.0)
Satisfaction with living allowance	
Yes	561 (50.9)
No	542 (49.1)
Satisfaction with standardized training	
Yes	961 (87.4)
No	139 (12.6)

### Professional identity and influencing factors

The mean total scores of the professional identity of the RTMSs were 3.58 ± 0.61, and the scores of the other dimension are listed in Table 3. The total scores of professional identity did not exhibit significant differences between the two groups (*P* = 0.058). In terms of cognition and emotionality dimension, the scores were significantly lower in the RTMSs than in the non-oriented medical students. Appendix 1 in the supplementary material shows the absolute frequency of ratings per item of the PIQUS.

**Table 3:** The scores of professional identity and DASS-21 of the participants

	RTMSs	Non-oriented medical students	*P* value
Professional identity			
Total scores	3.58 (0.61)	3.63 (0.67)	0.058
Cognition	3.65 (0.67)	3.72 (0.72)	0.007
Emotionality	3.63 (0.81)	3.74 (0.82)	0.002
Behavior	3.62 (0.68)	3.61 (0.78)	0.631
Fitness	3.35 (0.76)	3.35 (0.82)	0.994
DASS-21			
Depression	4.29 (4.06)	4.70 (4.55)	0.028
Anxiety	4.66 (3.85)	5.05 (4.51)	0.027
Stress	5.68 (4.20)	6.05 (4.74)	0.054

Data are expressed as Mean (SD)

In multiple linear regression analysis, Model 1 showed the following findings: junior college students were apparently inclined to a higher level of professional identity than undergraduate students (*β* = 0.054, *P* < 0.001); students at s clinical internship had a lower level of professional identity (*β* = − 0.169, *P* < 0.001); students with family monthly income of 5000 yuan or above were more likely to have lower level of professional identity (*β* = − 0.056, *P* = 0.023).

Model 2 showed that, compared with the students who enrolled in the RTME programme due to recommendations of family members or teachers, students whose policy choice motivation was to serve the local people (*β* = 0.174, *P* = 0.006) or personal interests (*β* = 0.032, *P* < 0.001) had a higher level of professional identity; nevertheless, those who enrolled in the RTME programme by mistake presented a lower level of professional identity (*β* = − 0.152, *P* = 0.007); compared with those who did not understand the policy prior to enrolment, students who generally understand the policy (*β* = 0.168, *P* = 0.004) or understanding the policy very or relatively well (*β* = 0.277, *P* < 0.001) were more inclined to a high level of professional identity; furthermore, students who understood the Healthy China 2030 Plan Outline were more likely to have higher level of professional identity than students who did not understand the policy; compared with those who believe that the RTME programme limits personal career development, students who considered that the programme can help achieve personal value (*β* = 0.296, *P* < 0.001) or offer a stable work (*β* = 0.188, *P* < 0.001) had higher level of professional identity. Model 3 further showed that, students who were unsatisfied with the six-year service period (*β* = − 0.124, *P* = 0.004), living allowance (*β* = − 0.117, *P* = 0.003), and standardized training (*β* = − 0.172, *P* = 0.001) were apparently inclined to a low level of professional identity (Table 4).

**Table 4:** Multiple linear regression on the RTMSs’ PIQUS^a^ total scores

Explanatory variables	Model1		Model 2		Model 3	
	*β* (Std. Error)	*P* value	*β* (Std. Error)	*P* value	*β* (Std. Error)	*P* value
Education level (Reference value:Undergraduate)						
Junior college	0.254 (0.041)	< 0.001	0.126 (0.041)	0.002	0.079 (0.041)	0.056
Academic years (Reference value: Pre-clinical)						
Clinical	−0.169 (0.041)	< 0.001	− 0.109 (0.039)	0.006	− 0.061 (0.039)	0.124
Family monthly income (Reference value: ≤5000)						
> 5000	−0.056 (0.024)	0.023	− 0.114 (0.044)	0.009	−0.094 (0.043)	0.029
Reasons for enrolment in the RTME programme (Reference value: Recommendations of family members or teachers)						
Economic reasons			−0.056 (0.063)	0.376	−0.049 (0.062)	0.432
Serve the local people			0.174 (0.063)	0.006	0.191 (0.061)	0.002
Guaranteed employment			−0.058 (0.067)	0.389	−0.061 (0.066)	0.360
Personal interests			0.232 (0.048)	< 0.001	0.220 (0.047)	< 0.001
By mistake			−0.152 (0.056)	0.007	−0.131 (0.055)	0.018
Understanding the conditions of policy before enrolling (Reference value: Don’t understand)						
Generally understand			0.168 (0.059)	0.004	0.142 (0.058)	0.014
Very or relatively well			0.277 (0.066)	< 0.001	0.221 (0.065)	< 0.001
Understanding the conditions of targeted primary health organization (Reference value: Don’t understand)						
Generally understand			0.032 (0.044)	0.468	0.033 (0.043)	0.445
Very or relatively well			−0.074 (0.064)	0.248	−0.081 (0.064)	0.201
Understand the Healthy China 2030 Plan Outline (Reference value: No)						
Yes			0.064 (0.069)	0.352	0.074 (0.068)	0.275
Understand the Equitable access to basic public health services (Reference value: No)						
Yes			0.132 (0.047)	0.005	0.100 (0.046)	0.029
View on the RTME programme (reference value: Limiting personal career development)						
Have a stable work			0.188 (0.039)	< 0.001	0.087 (0.041)	0.033
Achieve personal value			0.296 (0.067)	< 0.001	0.176 (0.068)	0.009
Other			0.213 (0.070)	0.002	0.157 (0.069)	0.024
Satisfaction with targeted primary health organization (Reference value: Yes)						
No					−0.060 (0.042)	0.156
Satisfaction with six years’ service period (Reference value: Yes)						
No					−0.124 (0.043)	0.004
Satisfaction with living allowance (Reference value: Yes)						
No					−0.117 (0.039)	0.003
Satisfaction with standardized training (Reference value: Yes)						
No					−0.172 (0.052)	0.001

*PIQUS* Professional Identity Questionnaire for Undergraduate Students

### Mental health and influencing factors

In our population, anxiety symptoms were highly prevalent among the RTMSs. The prevalence of depression, anxiety, and stress symptoms was at 11.8, 22.9 and 3.4%, correspondingly. In addition to stress, the scores of depression and anxiety were significantly lower in the RTMSs than in the non-oriented medical students. The multiple stepwise linear regression showed the following results: students with high level of professional identity were apparently inclined to better mental health; students involved in clinical internship were more likely to suffer from psychological disorders; junior college students were more inclined to suffer from depression (*β* = 1.457, *P* < 0.001) and anxiety (*β* = 1.375, *P* < 0.001) than undergraduate students; students who were unsatisfied with the targeted primary health organizations were more likely to suffer from depression (*β* = 0.522, *P* = 0.014) and anxiety (*β* = 0.529, *P* = 0.016). In addition, the students who applied for the RTME programme due to economic reason were more likely to suffer from anxiety than those who enrolled in the RTME programme due to recommendations of family members or teachers (*β* = 0.942, *P* = 0.013) (Table 5).

**Table 5:** Multiple stepwise linear regression of RTMSs’ mental health

	*β* (Std. error)	*P* value
Depression		
Professional identity	− 1.591 (0.181)	< 0.001
Education level (Reference value: Undergraduate)		
Junior college	1.457 (0.251)	< 0.001
Academic years (Reference value: Pre-clinical)		
Clinical	3.083 (0.240)	< 0.001
Satisfaction with targeted primary health organization (Reference value: Yes)		
No	0.552 (0.224)	0.014
Anxiety		
Professional identity	−1.169 (0.180)	< 0.001
Education level (Reference value: Undergraduate)		
Junior college	1.375 (0.247)	< 0.001
Academic years (Reference value: Pre-clinical)		
Clinical	2.689 (0.236)	< 0.001
Satisfaction with targeted primary health organization (Reference value: Yes)		
No	0.529 (0.218)	0.016
Reasons for enrolment in the RTME programme (Reference value: Recommendations of family members or teachers)		
Economic reason	0.942 (0.377)	0.013
Stress		
Professional identity	−1.355 (0.188)	< 0.001
Academic years (Reference value: Pre-clinical)		
Clinical	3.412 (0.237)	<0.001

## Discussion

### Professional identity in RTMSs

In terms of professional identity, no significant difference was found between RTMSs and non-oriented medical students. Nevertheless, our findings are inconsistent with other domestic studies, which showed that the RTMSs have a lower level of professional identity than the non-oriented medical students [25, 26]. These findings suggested that this phenomenon is frequently the result of personal, professional, and contextual factors, including heavy workload, lack of professional development space, geographic isolation, and concerns regarding educational, and employment opportunities for children. Given that all the RTMSs in Anhui Province were investigated, the result of the current study may have improved generalizability. Furthermore, in terms of cognition and emotionality dimension, the scores of the RTMSs were significantly lower than those of non-oriented medical students. According to the PIQUS, the dimension of cognition reflects the understanding of social value, career prospects and academic requirement of their major, and the dimension of emotionality reflects the satisfaction with their major, and whether they are willing to devote themselves to the profession after graduation. This result indicates that it’s essential for health professions educators to take measures to enhance the professional identity of RTMSs.

The need to connect theory with practice is an increasingly recognized aspect of medical education [27, 28]. Disjunction between the two may lead to the students feeling unrealistic and powerless. According to one study, students’ concepts of themselves as future doctors evolved quickly as they came into contact with patients [29]. Communications with other healthcare professionals also play an important role in the professional identity formation of the student doctors. For instance, the morning report, where students can directly interact with the senior doctors, is an excellent location for exchanging clinical experience and the popularization of medical knowledge [30]. One pedagogical approach that fosters the active involvement of the individual in the creation of their professional identity is narrative reflection. For trainee doctors, they could tell and retell the story of their experiences. Self-reflection of an individual’s thoughts, behavior and experience within their academic and clinical education promotes that person to develop their own understanding by which to lives as doctors [31]. With regard to the family economic factors, the students from the families with monthly income of more than 5000 yuan were found to be associated with weaken professional identity. This may be due to the fact that students from more wealthy family might give more priority to monetary income and that work in rural setting could not fulfill their will [32]. Many theoretical studies have suggested that intrinsic motivation strongly affects the job satisfaction of students [33, 34], however, in our study, only less than one-fifth of the RTMSs selected the programme because of personal interest. This study further verified this viewpoint. The students who selected the programme due to personal interests or serving the local people showed a higher level of professional identity than the students who enrolled in the programme because of recommendations of family members or teachers. Therefore, teachers and parents should fully respect the decisions of students and provide guidance on voluntarily applying for college entrance examination based on the personality traits and interests of students. In addition, students who have positive cognition of the RTME programme were more likely to devote themselves to primary healthcare service with high level of professional identity. The RTMSs who understand the policy of the RTME programme prior to enrolment or the equitable access to basic public health services were more likely to have a positive attitude towards the profession. Policy satisfaction (satisfaction with the six-year service period, living allowance, and standardized training) was significantly associated with professional identity. Therefore, the admission departments must strength the publicity of the RTME programme and introduce a specific content of the policy to students and parents in admission presentations to recruit high-quality students who aspire to serve the primary healthcare institutions. Meanwhile, the interview before enrolling can also be used to assess the applicants’ suitability as a supplementary measure, which may include their value, cognition, and other personality characteristics, so as to select qualified medical students who have a clear understanding of RTME policy and feel satisfied. Moreover, medical schools are responsible for conducting regular ideological and professional development education to enhance their cognition of current status and social value of rural healthcare, thus strengthening the noble mission and social responsibility for retaining primary healthcare workers.

### Mental health in RTMSs

In our study, the prevalence of depression, anxiety and stress were apparently lower than those in other studies using similar instruments among medical students in different countries [35–39]. Furthermore, the scores of depression and anxiety were significantly lower than those of non-oriented medical students, thereby indicating that the RTMSs have an improved psychological health status. For the RTMSs, we assumed that employment security, and the absence of academic financial burden may contribute to moderating the psychological distress. For example, a longitudinal population-based study demonstrated that exposure to poverty in childhood was found to be positively associated with many mental health problems in adolescence, such as depression [40]. Free tuition and a certain amount of living allowance during their studies relieves their family financial burden, and provides the economic support for professional learning. In addition, they need not worry about employment after graduation. To some extent, these factors may contribute to improving RTMSs’ mental health. However, additional studies on the prevalence of psychological distress in RTMSs are required to provide a comparison.

In the present study, students who underwent clinical training exhibit higher levels of psychological distress than those in pre-clinical years. This finding may be attributed to the contradiction between the desire to live in a metropolitan city and the reality of returning to primary healthcare institution after graduation. Moreover, the junior college students had a higher level of depression and anxiety than the undergraduate students. These students may feel inferior and worry that their knowledge is difficult to reconcile with job demand after entering the clinical practice. In addition, the satisfaction with targeted primary healthcare institutions was significantly associated with depression and anxiety. Therefore, strengthening the publicity of the RTME programme prior to enrolment and ensuring that these students can serve the local primary healthcare organizations may contribute to improving the mental health of the RTMSs. We also reported that students who enrolled in the RTME programme due to economic reason are likely to suffer from anxiety. For these students, the government could provide certain financial support to help them complete their studies and devote themselves to grassroots service after graduation with optimal psychological status.

This study showed statistically positive correlation between professional identity and mental health of the RTMSs. Our results indicated that the RTMSs with a higher level of professional identity has lower levels of psychological distress. By contrast, students who are dissatisfied with the profession may have increased negative emotions, such as complaints and tiredness in their study life, feelings of confusion for their future, or lacking explicit life plans, thereby making them likely to suffer from psychological disorders. Therefore, this study showed a new perspective which developing and improving professional identity may be beneficial for the RTMSs in promoting mental health.

### Strengths of the study

The current study is the first attempt to evaluate the professional identity and mental health in the RTMSs. The results of our study may be useful to the mental health prevention and promotion of professional identity for the RTMSs. Second, the sample size was adequate to assess the two constructs with favorable representation due to the investigating of all the RTMSs in Anhui Province. Third, we used DASS-21 to measure mental health problems and assessed the prevalence of depression, anxiety, and stress symptoms together. Finally, we found a positive association between professional identity and mental health of the RTMSs, thus indicating that subjects with low levels of professional identity may be susceptible to mental health problems.

### Limitations of the study

We recognized several important limitations. Considering that this study was based on the results obtained from a self-administrated questionnaire, recall bias should be included. Moreover, the results of students’ understanding of the RTME policy were based on self-reporting and were mostly subjective assessment. Although certain sociodemographic factors were included in the study, other factors, such as lifestyle and family history of psychological disorders, were disregarded. These factors should be considered in future research. Given that we conducted an observational study, no causal inferences can be drawn. Further longitudinal studies are necessary to elucidate the long-standing change in professional identity and mental health problems in this population.

## Conclusions

Given that the fundamental goal of medical education is to shape and cultivate students’ professional identity, appropriate strategies should be implemented. The policy publicity should be strengthened, and educators should improve the students’ cognition of the policy’s effectiveness and the social value of rural healthcare work, which could motivate students’ desire to perform rural healthcare work. Providing sound medical infrastructure and increasing remuneration in rural medical institutions may also benefit to the improvement of RTMSs’ professional identity. Furthermore, we also reported a positive association between students’ professional identity and mental health, this is a new perspective that shows that developing professional identity may prove helpful for medical students in promoting mental health.

## Appendix 1

**Table 6:** The absolute frequency of ratings per item of the professional identity Questionnaire for undergraduate students

Sub-dimension	Item	Ratings (%)		
		Inconformity	Neutral	Conformity
Cognition	I know the requirements for learners of the majors	9.7	21.2	69.1
	I know the employment situations of my major	12.8	24.1	63.1
	I know the status of my major in school	8.8	20.3	70.9
	I know the evaluation of my major outside	12.2	32.2	55.6
	I know the profession in general	8.0	19.5	72.5
Emotionality				
	I am willing to engage in job related to my major	10.3	18.7	71.1
	I have accepted my profession in my heart	10.3	17.9	71.9
	I have never thought about changing my major	20.8	18.3	60.9
	I have a positive evaluation of my major	9.4	16.2	74.3
	I have faith in the development prospect of my major	15.3	27.7	57.0
	I have a positive affection for my major.	13.2	28.2	58.6
	I am satisfied with the overall situation of my major	19.5	23.5	57.0
	I like my major in general	14.2	20.8	65.1
Behavior				
	I often read literature related to my major	31.3	27.0	41.7
	I often complete profession coursework in a timely and serious manner	8.0	13.2	78.9
	I can listen carefully to professional courses	6.6	12.9	80.5
	I spend a lot of time on professional learning	12.5	24.5	63.0
	I am persistent in studies of my major	11.0	22.9	66.2
	I actively participate in extracurricular activities related to the major	13.4	21.1	65.5
Fitness				
	I have a fine profession cogitation	10.3	37.9	51.8
	My disposition matches the major	12.8	30.9	56.4
	The major I have learned can reflect my strengths	15.2	42.9	41.9
	I feel at ease studying this major	29.7	32.4	37.9

## Appendix 2

To produce competent healthcare professionals for rural area, the Chinese government has launched a rural-oriented uition-waived medical education (RTME) programme since 2010. This programme aims to enroll medical students mainly from rural areas to work in township hospitals for 6 years after graduation. Medical universities and junior colleges are responsible for undertaking the education programme. The universities offer the 5 years medical education programme, while the junior colleges provide the 3 years medical education programme. Graduates of the 5-year programme earn a bachelor’s degree, and graduates of the 3-year programme receive an associate degree.

Each province which undertaking the rural-oriented tuition-waived medical education (RTME) programme has a quota every year. Applicants for the RTME programme should meet the following requirements: (1) students who graduates from high school are willing to devote themselves to the rural healthcare work; (2) the registered permanent residence of students and their legal guardians should be in the rural area, in addition, the students themselves ought to have local household registration for more than 3 consecutive years; (3) participants take the college entrance examination and obtain a qualified performance to meet the admission requirement of the RTME programme.

After admission, students are required to sign an employment agreement with the medical universities (colleges) and local health administrative department, promising that they will serve the local township hospitals for 6 years after graduation. The government would waive the related tuition fee for the students and provide them with living allowance during their school career in designated medical universities (colleges). Students who break the agreement will be refunded tuition that has been waived and compensate for the liquidated damages. The breach of contract will be recorded in the personal integrity file and physician integrity management.

## Abbreviations

DASS-21: Depression Anxiety Stress Scale-21
PIQUS: Professional Identity Questionnaire for Undergraduate Students
RTME: Rural-oriented Tuition-waived Medical Education
RTMS: Rural-oriented Tuition-waived Medical Student
